# Differential Expression of LncRNA in Bladder Cancer Development

**DOI:** 10.3390/diagnostics13101745

**Published:** 2023-05-15

**Authors:** Lorenzo Spirito, Rufina Maturi, Sara Carmela Credendino, Celeste Manfredi, Davide Arcaniolo, Marco De Martino, Francesco Esposito, Luigi Napolitano, Francesco Di Bello, Alfredo Fusco, Pierlorenzo Pallante, Marco De Sio, Gabriella De Vita

**Affiliations:** 1 Urology Unit, Department of Woman Child and General and Specialized Surgery, University of Campania “Luigi Vanvitelli”, Via De Crecchio 7, 80138 Naples, Italy; 2 Department of Molecular Medicine and Medical Biotechnology, University of Naples Federico II, Via Pansini 5, 80131 Naples, Italy; 3 Institute of Experimental Endocrinology and Oncology “G. Salvatore”, National Research Council (CNR), Via Pansini, 5, 80131 Naples, Italy; 4 Department of Precision Medicine, University of Campania “Luigi Vanvitelli”, Via De Crecchio 7, 80138 Napoli, Italy; 5 Urology Unit, Department of Neurosciences, Reproductive Sciences, and Odontostomatology, University of Naples Federico II, Via Pansini 5, 80131 Naples, Italy

**Keywords:** bladder cancer, lncRNAs, gene expression

## Abstract

Bladder cancer (BC) is the tenth most common cancer, with urothelial carcinoma representing about 90% of all BC, including neoplasms and carcinomas of different grades of malignancy. Urinary cytology has a significant role in BC screening and surveillance, although it has a low detection rate and high dependence on the pathologist’s experience. The currently available biomarkers are not implemented into routine clinical practice due to high costs or low sensitivity. In recent years, the role of lncRNAs in BC has emerged, even though it is still poorly explored. We have previously shown that the lncRNAs Metallophosphoesterase Domain-Containing 2 Antisense RNA 1 (MPPED2-AS1), Rhabdomyosarcoma-2 Associated Transcript (RMST), Kelch-like protein 14 antisense (Klhl14AS) and Prader Willi/Angelman region RNA 5 (PAR5) are involved in the progression of different types of cancers. Here, we investigated the expression of these molecules in BC, first by interrogating the GEPIA database and observing a different distribution of expression levels between normal and cancer specimens. We then measured them in a cohort of neoplastic bladder lesions, either benign or malignant, from patients with suspicion of BC undergoing transurethral resection of bladder tumor (TURBT). The total RNA from biopsies was analyzed using qRT-PCR for the expression of the four lncRNA genes, showing differential expression of the investigated lncRNAs between normal tissue, benign lesions and cancers. In conclusion, the data reported here highlight the involvement of novel lncRNAs in BC development, whose altered expression could potentially affect the regulatory circuits in which these molecules are involved. Our study paves the way for testing lncRNA genes as markers for BC diagnosis and/or follow-up.

## 1. Introduction

Bladder cancer (BC) is the tenth most commonly diagnosed cancer in the world population. The worldwide age-standardized BC incidence rate (per 100,000 persons/year) is 9.5 in men and 2.4 in women. Worldwide, the age-standardized BC mortality rate (per 100,000 persons/year) is 3.3 for men vs. 0.86 for women [1]. Approximately 75% of patients with BC present with a carcinoma in situ (CIS), defined as a high-grade flat lesion confined to the mucosa (stage Ta) or submucosa (stage T1); in younger patients (<40 years of age), this percentage is even higher [2]. Patients with Ta/T1 and CIS have a high prevalence due to long-term survival in many cases and a lower risk of cancer-specific mortality compared to T2-4 tumors [3]. Tobacco smoking is the most important risk factor for BC, accounting for approximately 50% of cases [2,4]. Occupational exposure to aromatic amines, polycyclic aromatic hydrocarbons and chlorinated hydrocarbons (industrial paint, metal and petroleum) is the second most important risk factor for BC, accounting for about 10% of all cases [5,6]. Exposure to ionizing radiation is connected with increased risk (3).

According to the WHO (2004), urothelial carcinoma, accounting for about 90% of all bladder cancers, includes papillary urothelial neoplasm of low malignant potential (PUNLMP) and non-invasive papillary carcinoma low grade (LG) and high grade (HG). The urinary cytology for exfoliated cancer cells has high sensitivity in HG tumors (84%) but low sensitivity in LG tumors (16%) [7]. The sensitivity of urine cytology in CIS detection is 28–100% [8]. However, while positive cytology indicates a urothelial carcinoma in the urinary tract, negative cytology does not exclude it. Numerous urinary tests have been developed, but none of these markers have been accepted as a routine practice by any clinical guidelines for diagnosis or follow-up. The diagnosis of papillary BC ultimately depends on cystoscopic examination of the bladder and histological evaluation of sampled tissue by either cold-cup biopsy or, most commonly, transurethral resection of bladder tumor (TURBT). The goal of TURBT is to make the correct histological diagnosis and staging and completely remove all visible lesions.

Long noncoding RNAs (lncRNAs) are noncoding transcripts at least 200 nucleotides long that are generally categorized into five groups based on their relative position on the DNA (sense, antisense, bidirectional, intronic and intergenic). They perform multiple functions, such as binding both to other nucleic acids (DNA or RNA) and proteins, and the possibility of folding into secondary structures allows them to form and/or stabilize multi-protein complexes [9] involved in signaling, scaffold, decoys and guides [10]. In this way, these molecules can control the expression of genes through different mechanisms, including the epigenetic modification of chromatin [11]. LncRNAs are involved in numerous physiological and pathological processes, including cancer, and they have been classified as oncogenes or tumor suppressors. Several of these molecules, indeed, have been characterized as useful biomarkers of both neoplastic onset and progression [12], and in recent years, the role of lncRNAs in bladder cancer has also emerged, although it is still unclear [13].

Our previous studies highlighted the involvement in the progression of different types of cancer of four lncRNAs: Metallophosphoesterase Domain-Containing 2 Antisense RNA 1 (MPPED2-AS1), Rhabdomyosarcoma-2 Associated Transcript (RMST), Kelch-like protein 14 antisense (Klhl14-AS) and Prader Willi/Angelman region RNA 5 (PAR5) [14,15,16,17].

MPPED2-AS1 has been shown to be downregulated in human thyroid and breast carcinoma samples, and we demonstrated that its restoration inhibited the proliferation and migration of thyroid carcinoma cell lines [14,18]. Interestingly, we found that the expression of MPPED2-AS1 was correlated with that of MPPED2, and we were able to demonstrate that MPPED2-AS1 can sustain the expression of MPPED2 [14,18]. It is well known that lncRNAs are able to interact with complexes methylating gene promoters and, interestingly, we demonstrated that MPPED2-AS1 could interact with DNA Methyltransferase 1 (DNMT1), blocking its activity on the MPPED2 promoter, thus allowing its protein expression [18].

The RMST lncRNA shows a key role in cell differentiation and the modulation of stemness since it is reported to interact with SRY-Box Transcription Factor 2 (SOX2), which is a master regulator of stem cell pluripotency [19,20]. We recently found it to be downregulated in anaplastic thyroid carcinomas [17], and our preliminary data show that RMST levels are strongly decreased in undifferentiated thyroid cancers. Intriguingly, we also found a simultaneous increase in SOX2 that inversely correlated with RMST in the same set of specimens, supporting a close relationship between RMST and SOX2, which is also reflected in their physical interaction.

Klhl14-AS has been described as a tumor suppressor in thyroid cancer, where its suppression correlates with the loss of differentiation markers and the decreased survival rate of thyroid follicular cells. KLHL14-AS acts as a competing endogenous RNA (ceRNA) in thyroid cancer by inhibiting the transforming activity of two oncogenic microRNAs [17]. Since Klhl14-AS, beyond its high expression levels in the thyroid gland, is expressed in several other human and mouse tissues, including the urinary bladder [21], we asked if it could also be involved in bladder carcinogenesis.

The lncRNA PAR5 has been recently characterized by our group as a tumor suppressor lncRNA acting through the interaction and inhibition of the Enhancer Of Zeste 2 (EZH2) protein in anaplastic thyroid cancer [17]. Interestingly, we demonstrated that PAR5 exerts its tumor suppressor role by inhibiting the activity of EZH2 on E-cadherin gene expression [17]. In fact, it is well known that EZH2 is able to epigenetically repress E-cadherin expression through the induction of H3K27 trimethylation at its promoter [22].

Here, we investigated the expression levels of these four tumor suppressor lncRNAs in bladder cancer, firstly by interrogating the GEPIA database to obtain evidence based on a large cohort of samples [23] and then validating and extending the in silico observations in a cohort of bladder neoplastic samples of different histological type and degree of malignancy by quantitative RT-PCR (qRT-PCR). In our cohort, we reveal differential expression levels of the analyzed lncRNAs between normal tissues, benign lesions and cancers. This analysis uncovers the involvement of novel lncRNAs that could be relevant in BC development, suggesting that the expression of lncRNAs in bladder cancer progression should be extensively analyzed to search for novel and more reliable disease markers.

## 2. Materials and Methods

### 2.1. Patient Enrollment, Sample Collection and Patient Evaluation

Consecutive patients undergoing TURBT at the University of Campania “Luigi Vanvitelli” hospital for suspicion of BC were included in the study. Patients undergoing TURBT or enucleation of the prostate for bladder outlet obstruction (BOO) were enrolled to obtain samples from non-cancerous bladders (healthy group). Subjects younger than 18 years of age, with a history of pelvic radiotherapy, undergoing adjuvant intravesical chemo- or immunotherapy, suffering from recurrent or chronic urinary tract infections (UTIs) or chronic primary pelvic pain syndrome (CPPPS), with preoperative positive urine culture, catheterized in the last 3 months, or having other concomitant cancers have been excluded from the study (both groups). Enrolled patients with suspicion of BC underwent TURBT with a monopolar 26-Ch resectoscope (Karl Storz, Tuttlingen, Germany) under spinal anesthesia. In the presence of multiple bladder lesions, the largest lesion has been chosen for the molecular tests. In the control group, only a single sample of an apparently healthy bladder was taken using a cold-cup biopsy. The samples were immediately frozen in liquid nitrogen and then stored at −80 °C and processed and analyzed. Each patient underwent a thorough medical history and physical examination.

### 2.2. In Silico LncRNA Gene Expression Analysis

The MPPED2-AS1, RMST, KLHL14-AS and PAR5 lncRNA expressions in BC tissues compared with their normal counterparts were investigated by Gene Expression Profiling Interactive Analysis (GEPIA, http://gepia.cancer-pku.cn/index.html, accessed on 26 January 2022), an online resource for cancer and normal gene expression profiling analysis based on the Cancer Genome Atlas (TCGA) database [23]. The RNA-Seq data for each gene were obtained from 404 BC tissues and 28 adjacent normal tissues.

### 2.3. LncRNA Gene Expression Analysis in Tissue Biopsies

The total RNA was isolated from specimens obtained from the University of Campania “Luigi Vanvitelli” using Trizol (Sigma Aldrich, St. Louis, MO, USA) reagent according to the manufacturer’s specifications. Following this, 1 μg of total RNA was used to obtain cDNA with the SensiFAST cDNA Synthesis Kit (BIOLINE BIO-65054), according to the manufacturer’s specifications. Quantitative real-time PCR on 20 ng of cDNA was performed with iTaq Universal SYBR Green Supermix (Bio-Rad, Hercules, CA, USA). Amplification cycles were carried out on CFX96 thermal cyclers (Biorad) in 96-well plates, and the raw data obtained were subsequently processed using the CFX Manager software (Biorad), which allows for obtaining the fold expression starting from the analysis of the threshold cycles. 18S has been used as an internal reference gene, and the fold change was calculated in pathological vs. non-pathological corresponding samples. The ΔCt method was applied for the analysis. The primer sequences are reported in Table 1.

### 2.4. Statistical Analysis

The expression data of the genes of interest correlated with the clinicopathological parameters of the patients, taking into account the benign or malignant nature of the lesions and the size of the malignant ones, considering 1 cm as the cut-off. GraphPad Prism 5.0 (GraphPad Software, Inc., San Diego, CA, USA) was used for the statistical analysis that was performed as follows: a comparison between the healthy group and each sample group was performed using Student’s *t*-test. A *p*-value less than 0.05 was considered statistically significant.

## 3. Results

### 3.1. In Silico Analysis of Gene Expression of the lncRNAs MPPED2-AS1, RMST, KLHL14-AS and PAR5 in Bladder Urothelial Cancer

**Figure 1:**
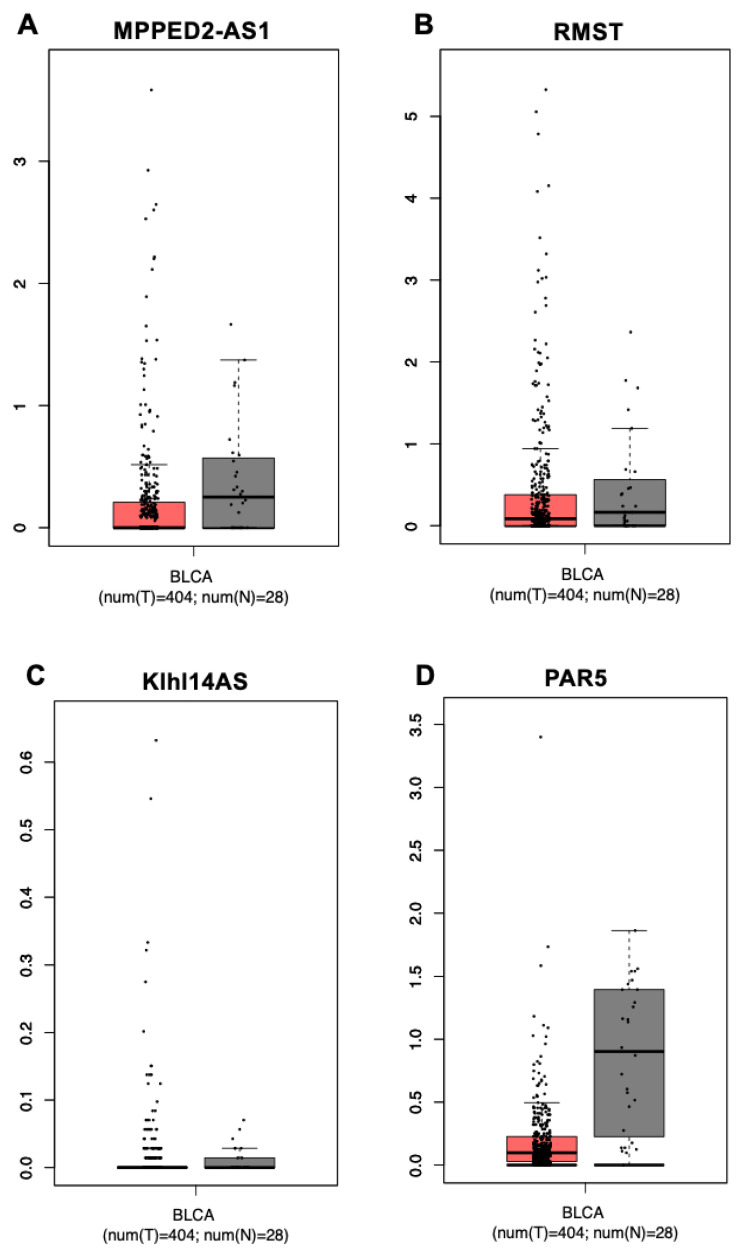
Expression of MPPED2-AS1, RMST, Klhl14AS and PAR5 lncRNAs in bladder urothelial carcinomas. Analysis of gene expression in the bladder cancer collection (BLCA) annotated in the GEPIA database. Box plot of MPPED2-AS1 (**A**), RMST (**B**), Klhl14-AS (**C**) and PAR5 (**D**) gene expression levels in 404 bladder urothelial carcinomas (T, red) and 28 normal bladders (N, grey).

Given the tumor suppressor role played by the lncRNAs MPPED2-AS1, RMST, Klhl14-AS and PAR5 in several types of cancers, where they are downregulated, we asked whether these lncRNAs could also be involved in bladder carcinogenesis. To analyze their expression levels in bladder cancer with respect to normal bladder tissue, we interrogated the GEPIA database, which reports a large dataset of bladder urothelial cancer (BLCA), composed of 404 cancer samples and 28 normal tissue controls [23]. For all of the analyzed lncRNAs, the median of the expression values, although to different extents, is lower in the bladder cancer samples than in the normal controls, even if these differences are not reported as statistically significant (Figure 1). It is worth noting, however, that the expression of MPPED2-AS1, RMST and Klhl14-AS is distributed over a much wider range of values in cancers than in normal tissues, while PAR5 values are scattered in a similar range between cancers and controls.

### 3.2. The lncRNAs MPPED2-AS1, RMST, Klhl14AS and PAR5 Show Differential Expression in Bladder Lesions of Different Degree of Malignancy

We then asked whether the downregulation observed in silico in carcinomas is a common event in bladder carcinogenesis or, alternatively, whether it is restricted to a specific subset of tumors. To answer this question, we collected biopsies from a cohort of 24 patients undergoing TURBT at our hospital for suspicion of BC. The enrolled patients were between 62 and 79 years old, the majority with a histological diagnosis of BLCA, showing a papillary growth pattern. Based on the results of the histological analysis, the biopsies were classified as benign lesions or bladder cancer, and cancers were divided into two groups depending on the size, smaller or larger than 1 cm. Cancer lesions showed a T classification value from T0 to T1, and no lymph node involvement nor metastatic spread was detected (Table 2). The healthy sample collection criteria are detailed in the Materials and Methods section.

**Figure 2:**
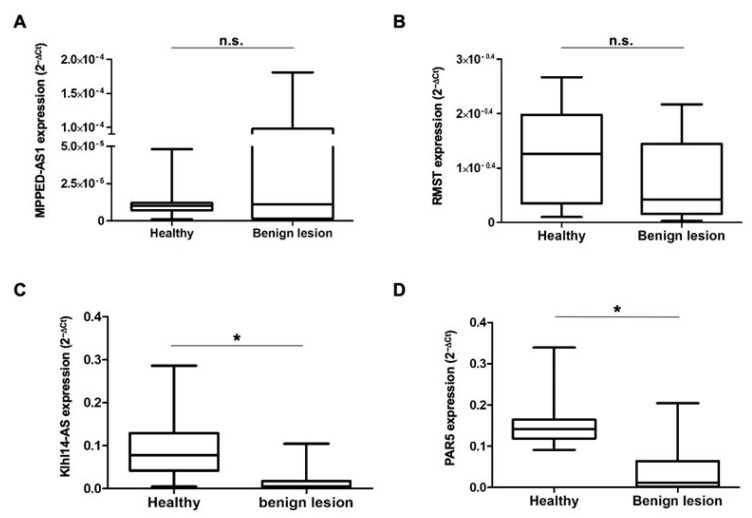
LncRNA expression in bladder benign lesions. Total RNA was isolated from bladder biopsies and lncRNAs levels were evaluated by qRT-PCR. Box plots of MPPED2-AS1 (**A**), RMST (**B**), Klhl14-AS (**C**) and PAR5 (**D**) expression levels, reported as 2^−ΔCt^, in healthy tissues and bladder benign lesions are shown. * Indicates *p*-value cut-off ≤ 0.05; n.s.-not significant.

The total RNA was extracted from each tumor and control sample, and the expression levels of the four lncRNAs were measured using qRT-PCR. We first compared the expression of each lncRNA in benign lesions to that in healthy controls. We found that, although the range of expression level values of MPPED2-AS1 is wider in the benign neoplastic samples than in the controls, the median value is similar in both groups (Figure 2A). For RMST, we observed a median value lower in benign lesions than in healthy controls, even if this difference is not statistically significant (Figure 2B). Klhl14AS and PAR5 expression levels, instead, show a dramatic and statistically significant reduction in benign lesions with respect to normal samples (Figure 2C,D).

**Figure 3:**
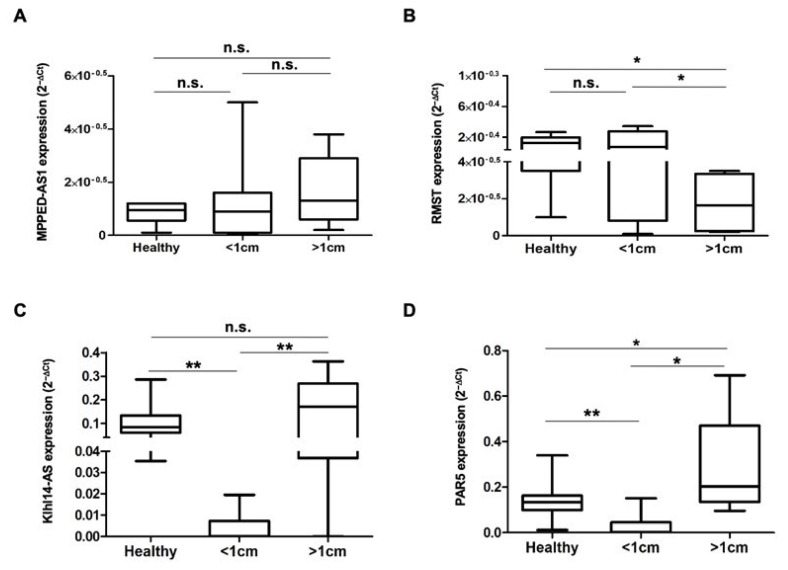
LncRNA expression in bladder malignant lesions. Total RNA was isolated from bladder biopsies and lncRNAs levels were evaluated by qRT-PCR. Box plots of MPPED2-AS1 (**A**), RMST (**B**), Klhl14-AS (**C**) and PAR5 (**D**) expression levels, reported as 2^−ΔCt^, in healthy tissues and bladder malignant lesions, grouped according to the size, are shown. * Indicates *p*-value cutoff ≤ 0.05; ** indicates *p*-value ≤ 0.01; n.s.—not significant.

Neoplastic bladder samples were then analyzed depending on their size by using 1 cm as a cut-off. As observed for benign lesions, the median value of MPPED2-AS1 expression levels is similar in both healthy samples and lesions, regardless of their size (Figure 3A). RMST analysis, instead, reveals that the expression of this lncRNA is not significantly altered in lesions smaller than 1 cm (even if the median value tends to be lower), while being significantly reduced in larger lesions both with respect to healthy controls and small lesions (Figure 3B). Figure 3C shows that Klhl14-AS expression levels in larger lesions is similar to that of the healthy samples, while it is dramatically and significantly reduced in lesions smaller than 1 cm, both with respect to healthy samples and large lesions. Similar to KLHL14-AS, PAR5 shows a significant and severe decrease in lesions smaller than 1 cm compared to the healthy controls, but its expression increases in larger lesions, in which the expression levels are even significantly higher than in the control samples (Figure 3D).

## 4. Discussion

During recent years, the need to find both early and prognostic markers for BC led researchers to focus on an emerging class of molecules, lncRNAs, which can act at several levels, from epigenetic to transcriptional or post-transcriptional regulation, thus affecting the onset, development and drug resistance of several types of cancer, including BC [13]. Here, we highlight for the first time the deregulation of three different lncRNAs, such as RMST, Klhl14-AS and PAR5, in bladder cancer samples of different histological types and degrees of malignancy.

All of the analyzed lncRNAs play tumor suppressor roles in other cancer types, and our analysis indicates that it could also be true in the case of bladder cancer. Here, we show that RMST shows a significant reduction only in big cancer lesions, with respect to both normal samples and lesions smaller than 1 cm. These data suggest that this lncRNA could play a tumor suppressor role in late steps of disease development, and its loss could be needed for BC progression towards high-grade disease. This observation is in agreement with recent findings reporting a dramatic decrease in RMST in anaplastic thyroid carcinomas, the most aggressive and undifferentiated type of human thyroid cancer [17]. On the contrary, Klhl14-AS and PAR5 are severely downregulated both in benign and small cancer lesions, while in large cancer lesions, their levels are rescued, suggesting that they could play a tumor suppressor role specifically in early phases of disease progression.

Interestingly, reduced PAR5 levels have also been confirmed in BC, and this occurrence lets us hypothesize that PAR5 could exert its functions via EZH2 to regulate its target genes in BC cells. In fact, it is known that EZH2, an enzymatic subunit of Polycomb Repressive Complex 2 (PRC2), promotes EMT through epigenetic repression of tumor suppressor genes by inducing tri-methylation of histone H3K27 (H3K27me3), and we already demonstrated that PAR5 facilitates the E-cadherin expression by contrasting the effect of EZH2 in thyroid cancer cells. It is likely that such a mechanism involving not only E-cadherin but also the epithelial cell adhesion molecule (EpCAM) gene could be active in BC, and this may be a stimulus for future studies concerning the involvement of PAR5 in this cancer type. Interestingly, in the literature, it is reported that E-cadherin and EpCAM genes are repressed by PRC2 [24,25], and, particularly, it has been found that in renal clear cell carcinomas and UTC, the EpCAM expression levels are associated with improved outcomes [24,26]. Consequently, the E-cadherin and EpCAM downregulation in BC and their connection with EZH2 could strongly represent an important node in this regulatory network.

Regarding the mechanism of action of these molecules, it is well known that many lncRNAs involved in carcinogenesis act as competing endogenous RNAs (ceRNAs): molecular sponges whose sequences share miRNA recognition sites with other RNAs [27]. Indeed, different studies identify the lncRNAs CASC11, CCAT1 and RMRP as BC tumor promoters acting as ceRNA [28,29,30]. Klhl14AS has already been described as ceRNA in thyroid cancer, where it acts as a decoy for miRNA182-5p and miRNA20a-5p, reducing their binding to the transcripts of both Pax8, a master regulator of thyroid differentiation, and Bcl2, a survival-promoting gene essential in thyroid physiology. Thus, Klhl14-AS decrease during tumor progression contributes to loss of differentiation and increased viability of thyroid cancer cells [16]. Here, we show that Klhl14-AS is strongly decreased in benign bladder neoplasms, while it is rescued in malignant lesions larger than 1 cm. This is different from our previous findings in thyroid cancers, where the expression of Klhl14-AS is inversely correlated with the increasing aggressiveness of cancers. We can speculate that, in the bladder, the decrease in Klhl14-AS could promote the early phases of tumor development, while the advanced cancers are no longer sensitive to its tumor suppressor activity. This hypothesis needs to be confirmed on a larger number of samples to verify if high Klhl14-AS expression in BC lesions could be considered a marker of advanced cancers. It is worth noting that Klhl14-AS is located on chromosome 18 in a head-to-head arrangement partially overlapping with the protein coding gene Klhl14, already shown to be involved in lymphoma and ovarian cancer [31,32,33]. Interestingly, this arrangement is preserved in different species [21], suggesting that it could be relevant to the function of these two genes. Indeed, many lncRNAs play their role in *cis* regulating neighbor genes by interacting directly with genomic DNA or with derived transcripts [34,35]. Although it is not yet proven that this is also true for the Klhl14 and Klhl14-AS gene pair, the observed changes in the expression of the lncRNA Klhl14-AS raise the question of whether the protein coding Klhl14 is also differentially expressed in BC.

The lncRNA RMST regulates the binding of SOX2 to its target promoters by physically interacting with it [19]. SOX2 is a transcription factor crucial for the maintenance of stem cell pluripotency, and its overexpression has been found in several cancer tissues and cells [20]. It is worth noting that SOX2 plays an important role also in the stemness of BC, where high levels of its mRNA are associated with high histological grade and advanced TNM stage, as well as shorter overall survival (OS) and disease-free survival (DFS) [36]. Interestingly, it has also been demonstrated that SOX2 overexpression induces fibronectin 1 (FN1) through the PI3K/AKT/NF-kB pathway in cancer [20]; therefore, it is not excluded that this occurrence could be valid also for BC. The observed decrease in RMST levels specifically in BC larger than 1 cm strongly suggests that it is linked to increased proliferation rate, decreased cell death or both. Further studies will be necessary to assess if the reduced RMST expression specifically observed in advanced lesions is functionally linked to the well-established role of SOX2 in BC progression.

The investigation of the altered expression of single lncRNAs in tumors has already identified several molecules such as MALAT1, HOTAIR and H19 as potential useful biomarkers for the diagnosis and prognosis of different types of cancer, including BC, because of their availability from body fluids, even if their mechanisms of action and hence their actual involvement in neoplastic transformation remain unclear [37,38]. It is worth noting that the development of molecular tools based on histological BC samples is of great interest for applicability in diagnosis and screening for similar research on urine and blood. To date, indeed, many studies have tried to find both markers or methodology to improve BC prevention, treatment and prognosis [39,40,41]. We aim to measure the expression of the analyzed lncRNAs in urine to explore the possibility of using these genes as noninvasive markers even before surgery to avoid unnecessary resection. In conclusion, this paper represents a basic study of the multiple steps required for the development of biomarkers. The next steps will be the validation of our data on an independent and large cohort of samples to verify the possibility of using a single or multiple noncoding gene signature as a BC marker.

## Figures and Tables

**Table 1:** Primers sequences used for qRT-PCR.

Gene	Forward Primer (5′ → 3′)	Reverse Primer (5′ → 3′)
18S	TGCGAGTACTCAACACCAACA	TTGGTGAGGTCAATGTCTGC
Klhl14-AS	CTTCGCTGCTGAGCTGAAAC	TTGTCATCTCTGTGAGACAGC
MPPED2-AS1	TGGTGCAGGGATTGTTGCAT	TGAACGACTGCAACTGCTTTG
RMST	GCCCAGCCCATTTCATTCAC	GCTCTGTCGTTCCACCTTGA
PAR5	GGTAGGAGGAGGGTTGGCTT	TTAGCAAGGCTGGACCTCAC

**Table 2:** Clinical data of patient cohort.

Gender	Male: 20 (83.3%) Female: 4 (16.7%)
Age	Years: 70.54 ± 7.82
Size	<1 cm: 13 (54.2%) ≥1 cm: 11 (45.8%)
Focality pattern	Monofocal: 17 (70.8%) Multifocal: 7 (29.2%)
Histology	Urothelial: 23 (95.8%) Squamous: 1 (4.2%)
Grade	Low: 18 (75.0%) High: 6 (25.0%)
Growth	Papillary: 20 (83.3%) Flat: 1 (4.2%) Solid: 3 (12.5%)
TNM	T0: 9 (37.5%) Ta:10 (41.7%) Tis:1 (4.2%) T1:4 (16.7%) T2-4: 0 (0%) N0: 24 (100%) M0: 24 (100%)

## Data Availability

The data presented in this study are available in figures of this article.
